# Detection and Characterization of RNA Viruses in Red Macroalgae (Bangiaceae) and Their Food Product (Nori Sheets)

**DOI:** 10.1264/jsme2.ME21084

**Published:** 2022-06-10

**Authors:** Yukino Mizutani, Yuto Chiba, Syun-ichi Urayama, Yuji Tomaru, Daisuke Hagiwara, Kei Kimura

**Affiliations:** 1 Analytical Research Center for Experimental Sciences, Saga University, Honjo-machi 1, Saga 840–8502, Japan; 2 Laboratory of Fungal Interaction and Molecular Biology (donated by IFO), Department of Life and Environmental Sciences, University of Tsukuba, 1–1–1 Tennodai, Tsukuba, Ibaraki, 305–8577, Japan; 3 Fisheries Technology Institute, Japan Fisheries Research and Education Agency, 2–17–5 Maruishi, Hatsukaichi, Hiroshima 739–0452, Japan; 4 Faculty of Agriculture, Saga University, Honjo-machi 1, Saga 840–8502, Japan

**Keywords:** RNA virus, red algae, *Neopyropia*, *Mitoviridae*, *Totiviridae*, fragmented and primer-ligated dsRNA sequencing, reverse transcription PCR

## Abstract

Persistent RNA viruses, which have been suggested to form symbiotic relationships with their hosts, have been reported to occur in eukaryotes, such as plants, fungi, and algae. Based on empirical findings, these viruses may also be present in commercially cultivated macroalgae. Accordingly, the present study aimed to screen red macroalgae (family Bangiaceae conchocelis and *Neopyropia yezoensis* thallus) and processed nori sheets (*N. yezoensis*) for persistent RNA viruses using fragmented and primer-ligated dsRNA sequencing (FLDS) and targeted reverse transcription PCR (RT-PCR). A *Totiviridae*-related virus was detected in the conchocelis of *Neoporphyra haitanensis*, which is widely cultivated in China, while two *Mitoviridae*-related viruses were found in several conchocelis samples and all *N. yezoensis*-derived samples (thallus and nori sheets). *Mitoviridae*-related viruses in* N. yezoensis* are widespread among cultivated species and not expected to inhibit host growth. *Mitoviridae*-related viruses were also detected in several phylogenetically distant species in the family Bangiaceae, which suggests that these viruses persisted and coexist in the family Bangiaceae over a long period of time. The present study is the first to report persistent RNA viruses in nori sheets and their raw materials.

Studies on viruses found in crop and ornamental plants as well as in humans have generally focused on pathogenicity (Scholthof *et al.*, 2011). However, recent metagenomic and metatranscriptomic ana­lyses revealed that plant viruses are more abundant than previously recognized and many inflict little or no harm on their hosts (Roossinck, 2012, 2015). Furthermore, certain RNA viruses are now considered to be “persistently transmitted” *via* cell-to-cell vertical transmission (Gray and Banerjee, 1999; Roossinck, 2010). Persistent infections by viruses in *Endornaviridae* and *Partitiviridae*, which infect the cytoplasm of plant cells, have been reported in a wide variety of crop species (*e.g.*, beans, wheat, rice, peppers, and fruits) and appear to cause no symptoms in their hosts (Roossinck, 2012). Due to the difficulties associated with the artificial transmission of persistent viruses to host cells, the functions of persistent viruses remain unclear.

Some RNA viruses, including persistent viruses, have been reported to benefit plants (Roossinck, 2011). Certain mosaic viruses, which cause the mottling of host plant leaves, were shown to confer drought or cold tolerance (Xu *et al.*, 2008). Furthermore, Jalapeño peppers (*Capsicum annuum*) infected with the persistent virus, *Pepper cryptic virus 1* (family *Partitiviridae*), were less susceptible to aphid feeding damage than virus-free jalapeño peppers (Safari *et al.*, 2019). The panic grass *Dichanthelium lanuginosum* requires the presence of the endophytic fungus *Curvularia protuberata* and the persistent fungal virus Curvularia thermal tolerance virus for survival in soils with temperatures higher than 50°C (Márquez *et al.*, 2007).

Persistent viruses have been documented in algae, although a number of pathogenic RNA viruses have also been detected (Short *et al.*, 2020; Sadeghi *et al.*, 2021). Viruses in *Partitiviridae* are persistent viruses in land plants that also occur in green algae (Ishihara *et al.*, 1992). In recent years, next-generation sequencing (NGS) revealed that viruses exist in algae without symptoms of viral infection, such as a persistent virus in the family *Endornaviridae*, which was detected in large brown macroalgae (Chiba *et al.*, 2020), and an RNA virus in the family* Totiviridae*, which was found in red macroalgae (Lachnit *et al.*, 2016; Chiba *et al.*, 2020). Although limited information is currently available on persistent viruses coexisting with algae, the great diversity of RNA viruses in marine environments suggests the existence of undiscovered viruses in macroalgae.

Therefore, cultivated macroalgae, such as crop plants, may form persistent and potentially beneficial relationships with viruses. The marine red algae *N. yezoensis* Ueda and *N. haitanensis* Chang et Zheng are cultivated in Japan, China, and Korea and are consumed worldwide. The many types of cultivated macroalgae in the family Bangiaceae, such as *N. yezoensis* and *N. haitanensis*, are farmed on a large scale and have recently been used globally to produce sushi and snack foods. Accordingly, the present study aimed to screen several species in the family Bangiaceae and nori sheets for persistent RNA viruses using fragmented and primer-ligated dsRNA sequencing (FLDS), which is an efficient method for thoroughly obtaining dsRNA sequences (Urayama *et al.*, 2016), and to investigate the spatiotemporal distribution of candidate persistent viruses in the family Bangiaceae using RT-PCR. This is the first study to document RNA viruses in commercially farmed macroalgae.

## Materials and Methods

### Sample collection

**Fig. 1.**
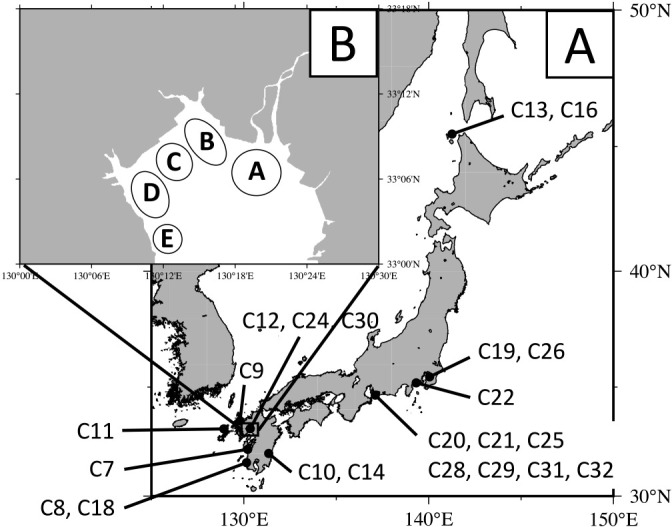
Red macroalgae (Bangiaceae) sampling locations. A, Conchocelis sampling locations in Japan. B, Thallus and nori sampling locations in the Ariake Sea.

The sample list is shown in Table 1. Nori sheets, which were made from the thalli of *N. yezoensis* cultivated at stations A–E in the Ariake Sea (Kyushu, Japan; Fig. 1), were purchased from or provided by each farmer and stored at –30°C for future ana­lyses. *N. yezoensis* thalli were collected from stations A, C, and D (Fig. 1) on a monthly basis between November 2018 and March 2019 and between December 2019 and February 2020. Thalli were not collected from station D in March 2019 because algae had not been harvested. Conchocelis samples collected from various locations in Japan (Table 1) were cultured in SWM-III medium (Ogata, 1970) at 18°C and with a 12-h photoperiod (30‍ ‍μmol photons‍ ‍m^–2^‍ ‍s^–1^) until RNA extraction.

### Comprehensive detection of RNA viruses in nori sheets and conchocelis samples using FLDS

Two nori sheets (N1 and N2) and six conchocelis samples (C1–6) were disrupted using liquid nitrogen in a mortar, and total nucleic acids were manually extracted from the samples using SDS-phenol. dsRNA was then purified using cellulose resin chromatography and subjected to agarose gel electrophoresis. To obtain sequence-grade dsRNA, the remaining DNA and ssRNA were removed using amplification-grade DNase I (Invitrogen) and S1 nuclease (Invitrogen).

The resulting sequence-grade dsRNA was converted into cDNA using the FLDS method (Urayama *et al.*, 2016; Hirai *et al.*, 2021). Briefly, dsRNA was fragmented using an ultrasonicator (Covaris S220; Covaris), adapter ligated (3′ ends), and converted to full-length cDNA using the SMARTer RACE 5′/3′ Kit (Takara Bio) with an adapter-complementary oligonucleotide primer. After PCR amplification, cDNA was fragmented using an ultrasonicator (Covaris S220), and Illumina sequencing libraries were constructed using the KAPA Hyper Prep Kit Illumina platforms (Kapa Biosystems). The resultant libraries were sequenced using the Illumina MiSeq v3 Reagent Kit (600 cycles) with 300-bp paired-end reads on the Illumina MiSeq platform.

### FLDS sequence ana­lysis

Raw dsRNA sequencing reads were processed as previously described (Hirai *et al.*, 2021). Briefly, adapter, low quality, low complexity, PhiX, and rRNA sequences were removed using a custom Perl script (https://github.com/takakiy/FLDS), and cleaned reads were subjected to *de novo* assembly using CLC Genomics Workbench (version 11.0; CLC Bio). Full-length sequences were obtained as previously described (Hirai *et al.*, 2021). BlastX (Camacho *et al.*, 2009) was used to identify similar protein sequences, and ORFs were identified and translated using ExPASy Translate (http://web.expasy.org/translate/), with the standard genetic code or the “mold, protozoan and coelenterate mitochondrial, mycoplasma/spiroplasma” genetic code. The RdRp amino acid sequences generated in the present study were aligned with their relative sequence in the NCBI database and the defined sequences by the International Committee on Taxonomy of Viruses (ICTV; https://talk.ictvonline.org/taxonomy/) using the FFT-NS-2 algorithm in MAFFT (v7.402; Katoh and Standley, 2013). To exclude ambiguous positions, the resulting sequence alignments were trimmed by trimAl (v1.4.rev15, ‘automated1’ mode; Capella-Gutiérrez *et al.*, 2009). Phylogenetic ana­lyses were performed using IQ-TREE, with ModelFinder to find the best-fit substitution model in each case and ultrafast nonparametric bootstrapping (1,000 replicates; Nguyen *et al.*, 2015). All phylogenic reconstructions were visualized using Figtree v1.4.4.

### Nested RT-PCR assay and sequencing of viruses in the family Bangiaceae conchocelis, *N. yezoensis* thallus, and nori sheets

*Mitoviridae*-related viruses and a *Totiviridae*-related virus obtained by FLDS were searched from the family Bangiaceae conchocelis, *N. yezoensis* thallus, and nori sheets using nested RT-PCR assays. Nested RT-PCR was performed on all samples in Table 1. Nori sheets were cut into small pieces, freeze-dried, and then pulverized using a Micro Smash MS-100 bead beater (TOMY). Thallus and conchocelis samples were frozen in liquid nitrogen and ground using a sterilized mortar and pestle. These homogenized samples for RNA extraction were suspended in TRIzol (Invitrogen) and stored at –80°C for future ana­lyses. RNA was extracted from algae samples using TRIzol (Invitrogen) and Phasemaker Tubes (Invitrogen) according to the manufacturer’s instructions. cDNA was synthesized from total RNA using SuperScript IV Reverse Transcriptase (Invitrogen) according to the manufacturer’s instructions.

Based on virus sequence data generated using FLDS, specific primer sets were designed using NCBI Primer-BLAST (http://www.ncbi.nlm.nih.gov/tools/primer-blast/), which yielded four primer pairs: NMV1-250F (5′-TAGAGGTTCCCAAGACATGG-3′) and NMV1-1610R (5′-AAGACTGGTCACCTTGTCTC-3′) for the first PCR targeting *Mitoviridae*-related sequence, NMV1-259F (5′-CCAAGACATGGTCTTGAGAG-3′) and NMV1-1476R (5′-AAGGAGGTCTTCAGAGACTC-3′) for the second PCR targeting the same *Mitoviridae*-related sequence as described above, NMV2-620F (5′-CTAGGAAGGAAGCCGACACC-3′) and NMV2-2188R (5′-GGCCGACAGTGGAATTAAGC-3′) for the first PCR targeting the other *Mitoviridae*-related sequence, NMV2-709F (5′-TCTTGACGCAAAGGCACTCT-3′) and NMV2-1946R (5′-GATCCGCCTGGAATCGTAGG-3′) for the second PCR targeting the second *Mitoviridae*-related sequence, NhaiTV1-716F (5′-TGTCTGGGAAATACGCCACC-3′) and NhaiTV1-2234R (5′-GATCTTGCCACCTCCAACCA-3′) for the first PCR targeting the *Totiviridae*-related sequence, and NhaiTV1-778F (5′-TCGGACAGCCTGGATGAGTA-3′) and NhaiTV1-1760R (5′-TTCCAACGTCACCGTCTCAC-3′) for the second PCR targeting the same sequence as the *Totiviridae*-related sequence as described above.

In the first PCR, the NMV1, NMV2, and NhaiTV1 sequences were amplified using 10-μL reaction mixtures that contained 1‍ ‍μL cDNA, 5‍ ‍μL EmeraldAmp PCR Master Mix (Takara Bio), and 0.5‍ ‍μM each of the corresponding forward and reverse primers. The following amplification conditions were used: initial denaturation at 98°C for 90 s; followed by 30 cycles at 98°C for 10‍ ‍s, at 60°C for 30‍ ‍s, and at 72°C for 1‍ ‍min; and a final extension at 72°C for 7‍ ‍min. The second PCR was performed using a 10-fold dilution of the primary amplicon as the template, the corresponding second PCR primers, and the same conditions used for the first PCR. The final PCR products were confirmed by electrophoresis, and notably, some samples with low amplification were subject to a third round of PCR by repeating the second PCR protocol.

The resulting nested PCR amplicons were sequenced using the BigDye Terminator v3.1 Cycle Sequencing Kit (Applied Biosystems), the corresponding second primer sets, and a Genetic Analyzer 3130 (Applied Biosystems). The appearances and lengths of the sequencing traces obtained were improved using PeakTrace (Nucleics Pty). Nucleotide sequence similarities between each sequence obtained were calculated using NCBI Blastn. Phylogenetic ana­lyses of partial NMV1 sequences (1,120 bp) were performed using the maximum likelihood (ML) method in MEGA7 v 7.0.26 (Kumar *et al.*, 2016) based on the Kimura 2-parameter model.

### Nested RT-PCR assay and sequencing of *Mitoviridae*-related viruses in protoplasts

To confirm that the detected viruses were derived from *Neopyropia* and not from the attached microbiomes, protoplasts were prepared from a thallus sample collected in November 2020, as described by Araki *et al.* (1987), and then washed with sterile seawater. Protoplasts were collected from pulverized samples by filtration. Nested RT-PCR assays and sequencing were performed according to the same method described above. The resulting suspension of protoplasts in sterile seawater was inoculated onto Marine agar 2216E (Difco) and incubated at 25°C for one week to confirm the absence of contaminating microbes.

### Species identification of conchocelis samples

To identify the species of conchocelis samples, the following experiments were conducted. DNA was extracted from ground conchocelis samples using DNAs-ici!-F (Rizo), and the large subunit of the ribulose bisphosphate carboxylase/oxygenase (*rbcL*) and 18S rRNA genes were sequenced as described by Yang *et al.* (2020). More specifically, *rbcL*-specific primers (*rbcL*-Rh1: 5′-AAGTGAACGTTACGAATCTG G-3′; rbcS1, 5′-AAAAGYYCCTTGTGTTARTCTCAC-3′; Hanyuda *et al.*, 2004) were used to amplify a 1,367-bp region of the *rbcL* gene, and 18S rRNA-specific primers (G06, 5′-GTTGGTGGTGCATGGCCGTTC-3′; Saunders and Kraft, 1994; G15.1, 5′-CTTGTTAGGACTTCTCCTTCC-3′; Müller *et al.*, 1998) to amplify the 520-bp V9 region of the 18S rRNA gene. PCR was performed using EmeraldAmp PCR Master Mix (Takara Bio) under the following conditions: at 98°C for 90 s; followed by 35 cycles at 98°C for 10‍ ‍s, at 55°C 30‍ ‍s, and at 72°C for 1‍ ‍min; and at 72°C for 10‍ ‍min. Sequencing was performed using the same conditions as those for NMV1 and NMV2 sequencing, and a ML phylogenetic tree was constructed using concatenated *rbcL* and 18S rRNA sequences from conchocelis samples and GenBank.

### Accession numbers

All sequences generated in the present study have been deposited in GenBank under the accession numbers LC660482–LC660594, LC660682–LC660689, LC698264, and DRA013081.

## Results

### FLDS and virus detection

FLDS-derived full-length sequences were attributed to three RNA viral genomes (Table 2). Two *Mitoviridae*-related sequences were named Neopyropia Mito-like virus 1 (NMV1, *Mitoviridae*) and Neopyropia Mito-like virus 2 (NMV2, *Mitoviridae*). These sequences were obtained from one of the conchocelis samples (C1), and NMV1 alone was detected in another conchocelis sample (C3). NMV1 and NMV2 were also detected in nori sheets samples (N1 and N2), which were made from the same species of thallus as C1. The *Totiviridae*-related sequence was named Neoporphyra haitanensis Toti-like virus 1 (NhaiTV1, *Totiviridae*). NhaiTV1 was detected in conchocelis sample C6. No viruses were detected in conchocelis sample C2, C4, or C5.

**Fig. 2.**
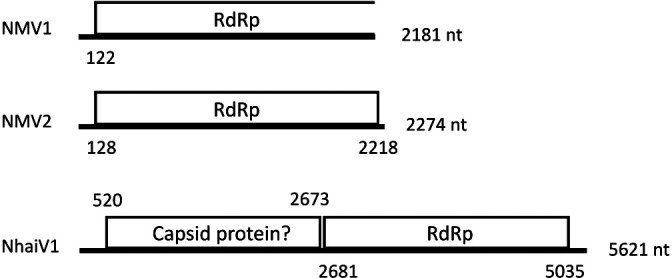
Genome structure of RNA viruses detected in species of Bangiaceae from Japan. Genome lengths are indicated on the right side of each genome illustration. The plus strand of each virus is depicted as a thick horizontal line.

**Fig. 3.**
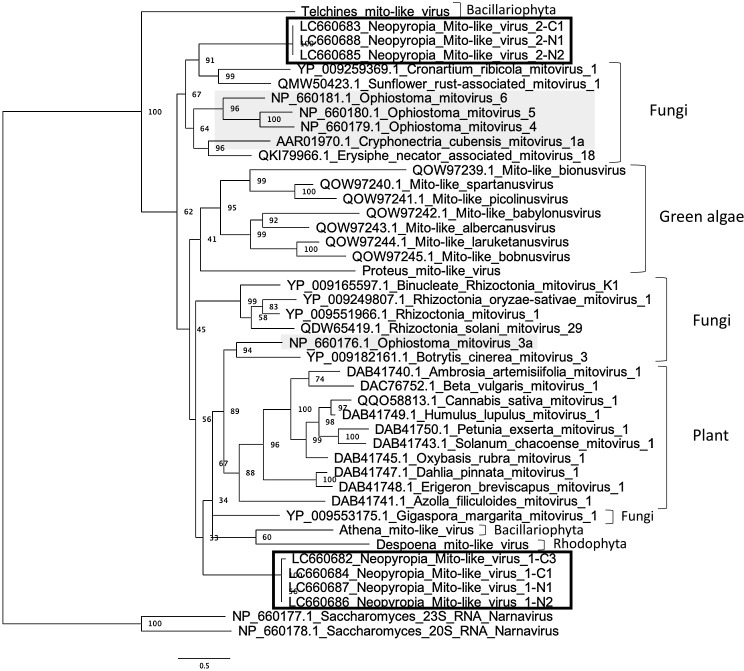
Phylogenetic tree of the family *Mitoviridae*. RdRp sequences were aligned using MAFFT 7.402 (FFT-NS-2), analyzed by ModelFinder to select the best-fit substitution model (LG+I+G4), and subjected to a phylogenetic ana­lysis using IQ-TREE and UFBoot as described in the Materials and Methods section. Bold boxes indicate viruses detected in the present study, and gray shading indicates ICTV-ratified species.

NMV1 and NMV2 both had unsegmented genomes, with only a single ORF, which encoded RdRp (Fig. 2). However, no stop codon was detected when the NMV1 genome was translated using the mold, protozoan, and coelenterate mitochondrial code or mycoplasma/spiroplasma genetic code. Based on blastx top hit sequences, the NMV1 and NMV2 amino acid sequences shared the most similarities with the sequences from Rhizoctonia mitovirus 1 (36.5–36.7% identity) and Erysiphe necator associated mitovirus 18 (33.0% identity), respectively. Based on pair-wise comparisons with the sequences defined by ICTV, the NMV1 and NMV2 amino acid sequences shared the most similarities with the sequences from *Ophiostoma mitovirus 3a* (32.4–33.1% identity) and *Ophiostoma mitovirus 5* (32.2% identity), respectively. A phylogenetic ana­lysis of RdRp sequences indicated that NMV1 and NMV2 formed clusters at different positions from each other; however, both sequences belonged to the family *Mitoviridae* (Fig. 3).

**Fig. 4.**
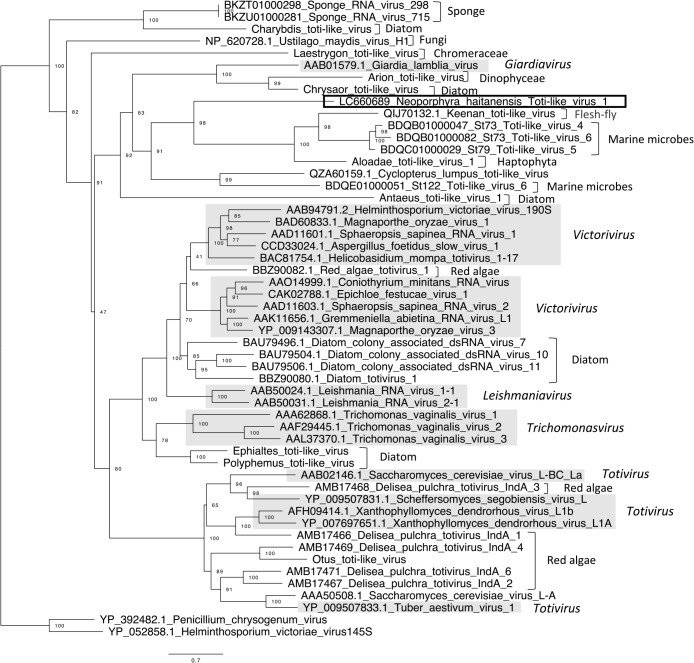
Phylogenetic tree of family *Totiviridae*. RdRp sequences were aligned using MAFFT 7.402 (FFT-NS-2), analyzed by ModelFinder to select the best-fit substitution model (LG+F+R5), and subjected to a phylogenetic ana­lysis using IQ-TREE and UFBoot as described in the Materials and Methods section. Bold boxes indicate viruses detected in the present study, and gray shading indicates ICTV-ratified species.

NhaiTV1 also had a non-segmented genome, but with two ORFs, a 5′-proximal ORF encoding an unknown protein and a 3′-proximal ORF encoding RdRp (Fig. 2). A Blastx search revealed that the RdRp amino acid sequence of NhaiTV shared the most similarities with the sequences from Keenan toti-like virus, which is associated with *Sarcophaga impatiens* (flesh flies; 30.2% identity). Among the sequences defined by ICTV, the RdRp amino acid sequence of NhaiTV was the most closely related to *Giardia lamblia virus* (genus *Giardiavirus*); however, sequence identity was low (26.32%). A phylogenetic ana­lysis of RdRp sequences indicated that NhaiTV1 formed a monophyletic group with *Giardiavirus* and sequences from marine microbes (Fig. 4). However, the length of the branch between NhaiTV1 and *Giardiavirus* was longer than that between *Victorivirus* and *Leishmaniavirus*, suggesting that at least NhaiTV1 is classified as a different genus from *Giardiavirus*.

### Distribution of identified viruses

**Fig. 5.**
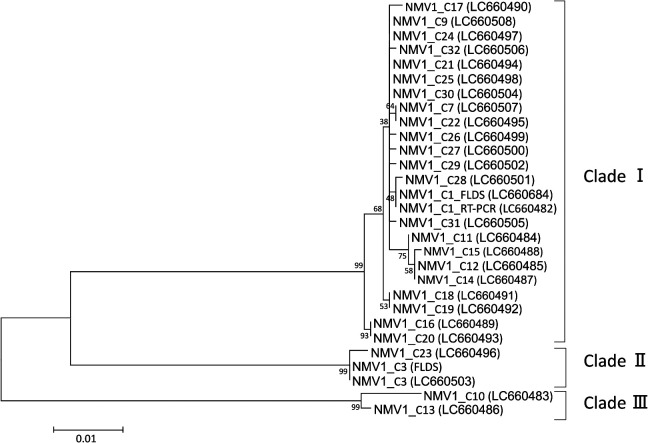
Maximum-likelihood tree of partial RdRp gene sequences from Neopyropia Mito-like virus 1 detected in Bangiaceae conchocelis samples. Node values indicate bootstrap support based on 1,000 replicates. The scale bar indicates the number of nucleotide substitutions per site.

Nested RT-PCR revealed that while NhaiTV1 was not detected in any samples, except for C6, NMV1 and NMV2 were both identified in all of the nori, thallus, and protoplast samples and in the majority of conchocelis samples (Table 3). However, NMV1 was not detected in C2, C29, C30, or C31, and neither NMV1 nor NMV2 was detected in C8, C11, and C28. Furthermore, nucleotide similarities between the NMV1 and NMV2 sequences obtained from conchocelis samples were 89.0–100% and 97.1–100%, respectively (Supplementary Table S1). The phylogenetic tree of NMV1 showed that each NMV1 sequence obtained from conchocelis samples was separated into three clades (I–III; Fig. 5), and the sequences within 1 clade showed pairwise nucleotide identities >97%. NMV1 sequences obtained from C3 and C23 were within clade II, those from C10 and C13 were within clade III, and all other sequences were within clade I. On the other hand, all NMV2 sequences were in one clade with identities >97%.

### Species identification in conchocelis samples

**Fig. 6.**
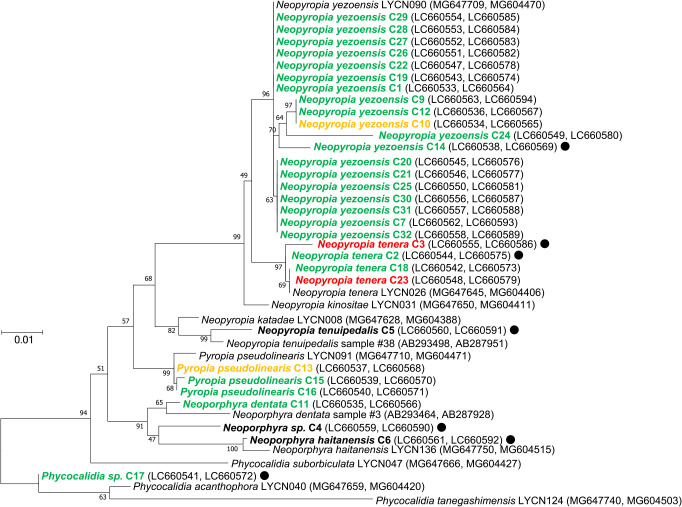
Maximum-likelihood tree of concatenated 18S rRNA and *rbcL* gene sequences from Bangiaceae specimens. Node values indicate bootstrap support based on 1,000 replicates. The scale bar indicates the number of nucleotide substitutions per site. Green, red, and yellow text indicate the detection of Neopyropia Mito-like virus 1 (NMV1) clades I, II, and III, respectively, whereas black text and black dots (●) indicate that NMV1 and NMV2, respectively, were not detected.

The phylogenetic tree based on concatenated 18S rRNA and *rbcL* gene sequences from the collected samples and GenBank indicated that each conchocelis sample was assignable to one out of three *Neopyropia* species, one *Pyropia* species, or two *Neoporphyra* species, except for C4 and C17, which did not cluster with any currently recognized species (Fig. 6). In the genus *Neopyropia*, the 18S rRNA and *rbcL* gene sequences of C1, C7, C9, C10, C12, C14, C19, C20, C21, C22, C24, C25, C26, C27, C28, C29, C30, C31, and C32 were clustered with *N. yezoensis*, those of C2, C3, C18, and C23 with *Neopyropia tenera*, and those of C5 with *N eopyropia tenuipedalis*. In the genus *Pyropia*, the concatenated sequences of C13, C15, and C16 were clustered with *Pyropia pseudolinearis*. In the genus *Neoporphyra*, *Neoporphyra dentata* and *Neoporphyra haitanensis* formed a cluster with C11 and C6, respectively. C8 was not identified with species because the sequence was duplicated.

## Discussion

The present study was conducted to test the hypothesis that macroalgae cultivated on a large scale also harbor persistent viruses. The results obtained revealed that several potential persistent RNA viruses were present in cultivated Bangiaceae and their food product, nori sheets. This is the first study to report RNA viruses in processed seafood products.

### *Totiviridae*-related virus in *N. haitanensis*

*Totiviridae*, which includes NhaiTV, is a group of dsRNA viruses with polycistronic mRNAs that encode capsid proteins (CP or Gag) and RdRp and currently includes five genera: *Totivirus*, *Victorivirus*, *Leishmaniavirus*, *Trichomonasvirus*, and *Giardiavirus* (Lefkowitz *et al.*, 2018; Walker *et al.*, 2020). Most the members of *Totiviridae* reside within the host cytoplasm and are generally transmitted through cell division, spore formation, or cell fusion. Members of the *Totiviridae* have been detected in a number of yeast, fungi, and human parasitic protozoa, as well as in various marine organisms, including microbial communities of surface seawater (Urayama *et al.*, 2018), microalgae (*e.g.*, Bacillariophyta, Dinophyceae, Haptophyta, and Rhodophyta; Urayama *et al.*, 2016; Chiba *et al.*, 2020; Charon *et al.*, 2021), and even sponges (Urayama *et al.*, 2020). These viruses have also been reported from other red macroalgae taxa, such as *Delisea pulchra* (Lachnit *et al.*, 2016) and *Pyropia suborbiculata* (Chiba *et al.*, 2020). Even though many of the members of *Totiviridae* have been reported in marine organisms, NhaiTV detected in the present study did not cluster with any of them, which suggests that NhaiTV represents a novel linage in *Totiviridae*. In addition, unique sequences, which are characteristic of the frame-shifting or stop/restart strategies used in the translation system of *Totiviridae*, were not found around the stop codon of ORF1 in the NhitTV1 genome (Brierley *et al.*, 1992; Powell, 2010; Li *et al.*, 2015; Atkins *et al.*, 2016; Jamal *et al.*, 2019). Furthermore, since we did not find this characteristic sequence in the genome of Keenan toti-like virus, which is closely related to NhitTV1, these viruses may use different translation strategies. Nested RT-PCR revealed that NhaiTV1 was only detected in C6, which was a conchocelis sample classified as *N. haitanensis*. NhaiTV1 was not detected in any other samples cultured by the same method as C6, suggesting that the virus is specific to this species or strain rather than to other organisms in seawater. One strategy to verify whether *N. haitanensis* is the host of NhaiTV1 is to use protoplasts from which adherent microorganisms have been removed. However, we did not confirm the host of NhaiTV1 using this approach because it was not possible to make protoplasts from the conchocelis. To rule out the possibility that NhaiTV1 is derived from the attached organisms, the localization of NhaiTV1 needs to be directly detected using morphological or other approaches.

### *Mitoviridae*-related viruses in *Neopyropia* spp.

The family *Mitoviridae* only comprises the genus *Mitovirus*, which is a genus of mitochondrial-localized (+) ssRNA viruses. *Mitovirus* cannot form capsids or envelopes for exposure to extracellular environments because their genomes only contain a single ORF encoding the RdRp gene (Polashock and Hillman, 1994). Therefore, mitoviruses exist as ribonucleoprotein complexes in host cells, instead of forming virions, and are transmitted *via* intracellular events, such as cell division and fusion (Polashock *et al.*, 1997; Giovannetti *et al.*, 1999). Mitoviruses were initially detected in the fungus *Cryphonectria parasitica* (Polashock and Hillman, 1994) and have only been reported from other fungal hosts. Additionally, although many plants endogenize the fragments of mitovirus genomes in their genome (Hong *et al.*, 1998; Bruenn *et al.*, 2015), mitoviruses had not been found in plants other than endogenized RNA virus elements until the study by Nibert *et al.* in 2018. These viruses were recently identified in green algae (Charon *et al.*, 2020) and microalgae (Charon *et al.*, 2021) in addition to plants. Although mitoviruses may have been detected from contaminated fungi instead of plants and algae considering the use of metagenomics, it is conceivable that mitoviruses infect fungi as well as a number of hosts, such as land plants and algae.

Phylogenetic ana­lyses showed that NMV1 and NMV2 formed clusters that were distinct from one another and also from other mitoviruses present in unicellular red and green algae. Therefore, NMV1 or NMV2 may have undergone a different speciation process from mitoviruses found in other algae. Since *Mitovirus* is only transmitted vertically, the phylogenetic tree of *Mitovirus* is expected to resemble that of its host. However, each mitovirus formed clusters in discrete locations, except for mitoviruses from plants or some green algae that clumped together and formed clusters in each. Current data on mitoviruses are insufficient to analyze distribution patterns; however, the placement of fungal mitoviruses between clusters of mitoviruses from plants or algae suggests that each host group (red algae, green algae, and land plants) has been independently infected *via* fungi.

NMV1 and NMV2 were both detected in all *N. yezoensis* samples and also in protoplasts, which ensures that these viruses were not derived from microbes attached to the surface of *N. yezoensis* and strongly suggests that they are maintained in *N. yezoensis*. These viruses were also detected in samples that were collected during different seasons or from different locations, and even in processed food samples, indicating that *N. yezoensis* is ubiquitously infected with NMV1 and NMV2 regardless of its life cycle phase, location, or season. In consideration of the mechanism of *Mitovirus* transmission, NMV1 and NMV2 appear to have been maintained in host cells since before speciation within the genus *Neopyropia* and they may have diversified alongside their hosts. This hypothesis is supported by the observation that NMV1 and NMV2 are evenly distributed throughout the genus. However, in the present study, these viruses were not detected in every *Neopyropia* specimen. Previous studies reported that *Endornavirus*, which is an RNA virus without a capsid or envelope, co-evolved with rice lineages and is not maintained when the host genome undergoes significant changes (Moriyama *et al.*, 1996; Horiuchi *et al.*, 2003; Fukuhara, 2019). In addition, the fungus *Cryphonectria parasitica* has been reported to transmit CpMV-1 dsRNA to only approximately 50% of its offspring if the maternal parent is infected at the time of mating (Polashock and Hillman, 1994).

*Mitoviridae* and most members of *Totiviridae* are vertically transmitted RNA viruses that lack an extracellular infection route, as observed in many persistent viruses. The functions of coexisting viruses currently remain unclear, and their effects on hosts require further investigation. Since *Mitoviridae*-related viruses were detected in all of the *N. yezoensis*-derived samples examined in the present study, our speculation is that these viruses are not parasitic and, instead, benefit their hosts. For example, *Totivirus* infecting the rice blast fungus *Magnaporthe oryzae* has been reported to activate secondary metabolism in its host (Ninomiya *et al.*, 2020), and *Narnavirus*, a close relative of *Mitovirus*, has been shown to play a role in sexual reproduction by its host (Espino-Vázquez *et al.*, 2020). Difficulties are associated with controlling infections by viruses that are not shed from cells, particularly *Mitoviridae*. However, overcoming this hurdle will facilitate investigations on the relationships between persistent viruses and their hosts and, thus, the future use of persistent viruses in agricultural and aquacultural breeding programs.

## Citation

Mizutani, Y., Chiba, Y., Urayama, S., Tomaru, Y., Hagiwara, D., and Kimura, K. (2022) Detection and Characterization of RNA Viruses in Red Macroalgae (Bangiaceae) and Their Food Product (Nori Sheets). *Microbes Environ ***37**: ME21084.

https://doi.org/10.1264/jsme2.ME21084

## Supplementary Material

Supplementary Material

## Figures and Tables

**Table 1.** List of algal samples.

Conchocelis			Thallus				Nori sheets		
Sample ID	Collection source		Sample ID	Collection date (M/Y)	Collection site		Sample ID	Collection date (M/D/Y)	Collection site
C1	The same sample as Pyr_1 in Nagano *et al.*, 2021		T1	11/2018	Station A, Ariake Sea, Japan		N1	11/21/2017	Station B, Ariake Sea, Japan
C2	The same sample as Pyr_19 in Nagano *et al.*, 2021		T2	12/2018	Station A, Ariake Sea, Japan		N2	11/21/2017	Station B, Ariake Sea, Japan
C3	The same sample as Pyr_27 in Nagano *et al.*, 2021		T3	01/2019	Station A, Ariake Sea, Japan		N3	11/21/2017	Station A, Ariake Sea, Japan
C4	The same sample as Pyr_35 in Nagano *et al.*, 2021		T4	02/2019	Station A, Ariake Sea, Japan		N4	01/08/2018	Station E, Ariake Sea, Japan
C5	The same sample as Pyr_44 in Nagano *et al.*, 2021		T5	03/2019	Station A, Ariake Sea, Japan		N5	01/08/2018	Station A, Ariake Sea, Japan
C6	The same sample as Pyr_45 in Nagano *et al.*, 2021		T6	12/2019	Station A, Ariake Sea, Japan		N6	01/29/2018	Station C, Ariake Sea, Japan
C7	Ashikita, Kumamoto		T7	01/2020	Station A, Ariake Sea, Japan		N7	02/06/2018	Station D, Ariake Sea, Japan
C8	Izumi, Kagoshima		T8	02/2020	Station A, Ariake Sea, Japan		N8	02/08/2018	Station E, Ariake Sea, Japan
C9	Matsuura, Fukushima		T9	11/2018	Station C, Ariake Sea, Japan		N9	02/08/2018	Station C, Ariake Sea, Japan
C10	Matsushima, Miyazaki		T10	12/2018	Station C, Ariake Sea, Japan		N10	02/09/2018	Station B, Ariake Sea, Japan
C11	Uku Island, Nagasaki		T11	01/2019	Station C, Ariake Sea, Japan		N11	02/09/2018	Station C, Ariake Sea, Japan
C12	Ariake sea, Fukuoka		T12	02/2019	Station C, Ariake Sea, Japan		N12	02/09/2018	Station D, Ariake Sea, Japan
C13	Rebun Island, Hokkaidō		T13	03/2019	Station C, Ariake Sea, Japan		N13	02/09/2018	Station C, Ariake Sea, Japan
C14	Matsushima, Miyazaki		T14	12/2019	Station C, Ariake Sea, Japan		N14	02/09/2018	Station B, Ariake Sea, Japan
C15	Hokkaidō		T15	01/2020	Station C, Ariake Sea, Japan		N15	02/14/2018	Station C, Ariake Sea, Japan
C16	Rebun Island, Hokkaidō		T16	02/2020	Station C, Ariake Sea, Japan		N16	02/22/2018	Station D, Ariake Sea, Japan
C17	Urasoe, Okinawa		T17	11/2018	Station D, Ariake Sea, Japan		N17	02/26/2018	Station B, Ariake Sea, Japan
C18	Izumi, Kagoshima		T18	12/2018	Station D, Ariake Sea, Japan		N18	02/28/2018	Station C, Ariake Sea, Japan
C19	Narawa, Chiba		T19	01/2019	Station D, Ariake Sea, Japan		N19	11/29/2018	Station C, Ariake Sea, Japan
C20	Noma, Aichi		T20	02/2019	Station D, Ariake Sea, Japan		N20	12/02/2018	Station C, Ariake Sea, Japan
C21	Noma, Aichi		T21	12/2019	Station D, Ariake Sea, Japan		N21	12/02/2018	Station A, Ariake Sea, Japan
C22	Odawara, Kanagawa		T22	01/2020	Station D, Ariake Sea, Japan		N22	01/11/2019	Station A, Ariake Sea, Japan
C23	Artificial hybrids, Japan		T23	02/2020	Station D, Ariake Sea, Japan		N23	01/19/2019	Station A, Ariake Sea, Japan
C24	Ariake sea, Fukuoka		T24P	11/2020	Ariake Sea, Japan		N24	01/26/2019	Station A, Ariake Sea, Japan
C25	Noma, Aichi						N25	01/27/2019	Station C, Ariake Sea, Japan
C26	Narawa, Chiba						N26	01/28/2019	Station B, Ariake Sea, Japan
C27	Dalian, China						N27	02/02/2019	Station A, Ariake Sea, Japan
C28	Noma, Aichi						N28	02/08/2019	Station A, Ariake Sea, Japan
C29	Noma, Aichi						N29	02/08/2019	Station B, Ariake Sea, Japan
C30	Ariake sea, Saga						N30	02/16/2019	Station A, Ariake Sea, Japan
C31	Noma, Aichi						N31	02/25/2019	Station A, Ariake Sea, Japan
C32	Noma, Aichi						N32	03/11/2019	Station D, Ariake Sea, Japan
							N33	03/11/2019	Station D, Ariake Sea, Japan
							N34	03/11/2019	Station D, Ariake Sea, Japan

**Table 2.** List of viruses obtained by FLDS

Virus	Detection source/ Likely host	The best hit sequence on a blastx search			The best hit sequence on ICTV-registered species	
		Sequence name/ host	Identity (%)		Species name/ host	Identity (%)
NMV1	N1/*N. yezoensis*	Rhizoctonia mitovirus 1/ *Rhizoctonia solani*	36.7		*Ophiostoma mitovirus 3a/ Ophiostoma novo-ulmi*	33.1
NMV1	N2/*N. yezoensis*	Rhizoctonia mitovirus 1/ *Rhizoctonia solani*	36.7		*Ophiostoma mitovirus 3a/ Ophiostoma novo-ulmi*	33.1
NMV1	C1/*N. yezoensis*	Rhizoctonia mitovirus 1/ *Rhizoctonia solani*	36.7		*Ophiostoma mitovirus 3a/ Ophiostoma novo-ulmi*	33.1
NMV1	C3/*N. tenera*	Rhizoctonia mitovirus 1/ *Rhizoctonia solani*	36.5		*Ophiostoma mitovirus 3a/ Ophiostoma novo-ulmi*	32.4
NMV2	N1/*N. yezoensis*	Erysiphe necator associated mitovirus 18/ *Erysiphe necator*	33.0		*Ophiostoma mitovirus 5/ Ophiostoma novo-ulmi*	32.2
NMV2	N2/*N. yezoensis*	Erysiphe necator associated mitovirus 18/ *Erysiphe necator*	33.0		*Ophiostoma mitovirus 5/ Ophiostoma novo-ulmi*	32.2
NMV2	C1/*N. yezoensis*	Erysiphe necator associated mitovirus 18/ *Erysiphe necator*	33.0		*Ophiostoma mitovirus 5/ Ophiostoma novo-ulmi*	32.2
NhaiTV1	C6/*N. haitanensis*	Keenan toti-like virus/ *Sarcophaga impatiens*	30.2		*Giardia lamblia virus/ Giardia lamblia*	26.3

**Table 3.** Detection of *Mitoviridae*-related viruses and a *Totiviridae*-related virus in algal samples. The species identification of conchocelis samples is based on the results of Fig. 6. Note: +, positive; w, weak (*i.e.*, PCR products obtained, but the bands were very faint); –, negative.

Sample ID	Species	NMV1	NMV2	NhaiTV1		Sample ID	Species	NMV1	NMV2	NhaiTV1
C1	*N. yezoensis*	+	+	–		C18	*N. tenera*	+	+	–
C2	*N. tenera*	–	–	–		C19	*N. yezoensis*	+	+	–
C3	*N. tenera*	+	–	–		C20	*N. yezoensis*	+	+	–
C4	*Neoporphyra* sp.	–	–	–		C21	*N. yezoensis*	+	+	–
C5	*N. tenuipedalis*	–	–	–		C22	*N. yezoensis*	+	+	–
C6	*N. haitanensis*	–	–	+		C23	*N. tenera*	+	+	–
C7	*N. yezoensis*	w+	+	–		C24	*N. yezoensis*	+	+	–
C8	Contaminated	–	–	–		C25	*N. yezoensis*	w+	+	–
C9	*N. yezoensis*	w+	+	–		C26	*N. yezoensis*	+	+	–
C10	*N. yezoensis*	+	+	–		C27	*N. yezoensis*	+	+	–
C11	*N. dentata*	w+	+	–		C28	*N. yezoensis*	+	+	–
C12	*N. yezoensis*	+	+	–		C29	*N. yezoensis*	w+	+	–
C13	*P. pseudolinearis*	w+	+	–		C30	*N. yezoensis*	+	+	–
C14	*N. yezoensis*	w+	–	–		C31	*N. yezoensis*	+	+	–
C15	*P. pseudolinearis*	w+	+	–		C32	*N. yezoensis*	+	+	–
C16	*P. pseudolinearis*	w+	+	–		T1-T24P		+	+	–
C17	*Phycocalidia sp.*	w+	–	–		N1-N34		+	+	–

## References

[B1] Araki, T., Aoki, T., and Kitamikado, M. (1987) Preparation and regeneration of protoplasts from wild-type of *Porphyra yezoensis* and green variant of *P. tenera*. Nippon Suisan Gakkaishi 53: 1623–1627 (in Japanese).

[B2] Atkins, J.F., Loughran, G., Bhatt, P.R., Firth, A.E., and Baranov, P.V. (2016) Ribosomal frameshifting and transcriptional slippage: From genetic steganography and cryptography to adventitious use. Nucleic Acids Res 44: 7007–7078. 27436286 10.1093/nar/gkw530 PMC5009743

[B3] Brierley, I., Jenner, A.J., and Inglis, S.C. (1992) Mutational ana­lysis of the “slippery-sequence” component of a coronavirus ribosomal frameshifting signal. J Mol Biol 227: 463–479. 1404364 10.1016/0022-2836(92)90901-U PMC7125858

[B4] Bruenn, J.A., Warner, B.E., and Yerramsetty, P. (2015) Widespread *mitovirus* sequences in plant genomes. PeerJ 3: e876. 25870770 10.7717/peerj.876 PMC4393810

[B5] Camacho, C., Coulouris, G., Avagyan, V., Ma, N., Papadopoulos, J., Bealer, K., and Madden, T.L. (2009) BLAST+: architecture and applications. BMC Bioinf 10: 1–9. 10.1186/1471-2105-10-421 PMC2803857 20003500

[B6] Capella-Gutiérrez, S., Silla-Martínez, J.M., and Gabaldón, T. (2009) trimAl: a tool for automated alignment trimming in large-scale phylogenetic ana­lyses. Bioinformatics 25: 1972–1973. 19505945 10.1093/bioinformatics/btp348 PMC2712344

[B7] Charon, J., Marcelino, V.R., Wetherbee, R., Verbruggen, H., and Holmes, E.C. (2020) Metatranscriptomic identification of diverse and divergent RNA viruses in green and Chlorarachniophyte algae cultures. Viruses 12: 1180. 33086653 10.3390/v12101180 PMC7594059

[B8] Charon, J., Murray, S., and Holmes, E.C. (2021) Revealing RNA virus diversity and evolution in unicellular algae transcriptomes. Virus Evol 7: 1–18. 10.1093/ve/veab070 PMC9927876 36819971

[B9] Chiba, Y., Tomaru, Y., Shimabukuro, H., Kimura, K., Hirai, M., Takaki, Y., et al. (2020) Viral RNA genomes identified from marine macroalgae and a diatom. Microbes Environ 35: ME20016. 32554943 10.1264/jsme2.ME20016 PMC7511793

[B10] Espino-Vázquez, A.N., Bermúdez-Barrientos, J.R., Cabrera-Rangel, J.F., Córdova-López, G., Cardoso-Martínez, F., Martínez-Vázquez, A., et al. (2020) Narnaviruses: novel players in fungal–bacterial symbioses. ISME J 14: 1743–1754. 32269378 10.1038/s41396-020-0638-y PMC7305303

[B11] Fukuhara, T. (2019) Endornaviruses: persistent dsRNA viruses with symbiotic properties in diverse eukaryotes. Virus Genes 55: 165–173. 30644058 10.1007/s11262-019-01635-5

[B12] Giovannetti, M., Azzolini, D., and Citernesi, A.S. (1999) Anastomosis formation and nuclear and protoplasmic exchange in arbuscular mycorrhizal fungi. Appl Environ Microbiol 65: 5571–5575. 10584019 10.1128/aem.65.12.5571-5575.1999 PMC91759

[B13] Gray, S.M., and Banerjee, N. (1999) Mechanisms of arthropod transmission of plant and animal viruses. Microbiol Mol Biol Rev 63: 128–148. 10066833 10.1128/mmbr.63.1.128-148.1999 PMC98959

[B51] Hanyuda, T., Suzawa, Y., Suzawa, T., Arai, S., Sato, H., Ueda, K., and Kumano, S. (2004) Biogeography and taxonomy of *Batrachospermum helminthosum* (Batrachospermales, Rhodophyta) in Japan inferred from *rbcL* gene sequences. J Phycol 40: 581–588.

[B14] Hirai, M., Takaki, Y., Kondo, F., Horie, M., Urayama, S.I., and Nunoura, T. (2021) RNA viral metagenome ana­lysis of subnanogram dsRNA using fragmented and primer ligated dsRNA sequencing (FLDS). Microbes Environ 36: ME20152. 33952860 10.1264/jsme2.ME20152 PMC8209451

[B15] Hong, Y., Cole, T.E., Brasier, C.M., and Buck, K.W. (1998) Evolutionary relationships among putative RNA-dependent RNA polymerases encoded by a mitochondrial virus-like RNA in the Dutch elm disease fungus, *Ophiostoma novo-ulmi,* by other viruses and virus-like RNAs and by the *Arabidopsis* mitochondrial genome. Virology 246: 158–169. 9657003 10.1006/viro.1998.9178

[B16] Horiuchi, H., Moriyama, H., and Fukuhara, T. (2003) Inheritance of *Oryza sativa* *endornavirus* in F1 and F2 hybrids between japonica and indica rice. Genes Genet Syst 78: 229–234. 12893964 10.1266/ggs.78.229

[B17] Ishihara, J., Pak, J.Y., Fukuhara, T., and Nitta, T. (1992) Association of particles that contain double-stranded RNAs with algal chloroplasts and mitochondria. Planta 187: 475–482. 24178141 10.1007/BF00199965

[B18] Jamal, A., Sato, Y., Shahi, S., Shamsi, W., Kondo, H., and Suzuki, N. (2019) Novel *Victorivirus* from a Pakistani isolate of *Alternaria alternata* lacking a typical translational stop/restart sequence signature. Viruses 11: 577. 31242660 10.3390/v11060577 PMC6631646

[B19] Katoh, K., and Standley, D.M. (2013) MAFFT multiple sequence alignment software version 7: Improvements in performance and usability. Mol Biol Evol 30: 772–780. 23329690 10.1093/molbev/mst010 PMC3603318

[B20] Kumar, S., Stecher, G., Tamura, K., and Dudley, J. (2016) MEGA7: molecular evolutionary genetics ana­lysis version 7.0 for bigger datasets. Mol Biol Evol 33: 1870–1874. 27004904 10.1093/molbev/msw054 PMC8210823

[B21] Lachnit, T., Thomas, T., and Steinberg, P. (2016) Expanding our understanding of the seaweed holobiont: RNA viruses of the red alga *Delisea pulchra*. Front Microbiol 6: 1489. 26779145 10.3389/fmicb.2015.01489 PMC4705237

[B22] Lefkowitz, E.J., Dempsey, D.M., Hendrickson, R.C., Orton, R.J., Siddell, S.G., and Smith, D.B. (2018) Virus taxonomy: the database of the international committee on taxonomy of viruses (ICTV). Nucleic Acids Res 46: D708–D717. 29040670 10.1093/nar/gkx932 PMC5753373

[B23] Li, H., Havens, W.M., Nibert, M.L., and Ghabrial, S.A. (2015) An RNA cassette from *Helminthosporium victoriae* virus 190S necessary and sufficient for stop/restart translation. Virology 474: 131–143. 25463611 10.1016/j.virol.2014.10.022

[B25] Márquez, L.M., Redman, R.S., Rodriguez, R.J., and Roossinck, M.J. (2007) A virus in a fungus in a plant: three-way symbiosis required for thermal tolerance. Science 315: 513–515. 17255511 10.1126/science.1136237

[B26] Moriyama, H., Kanaya, K., Wang, J.Z., Nitta, T., and Fukuhara, T. (1996) Stringently and developmentally regulated levels of a cytoplasmic double-stranded RNA and its high-efficiency transmission via egg and pollen in rice. Plant Mol Biol 31: 713–719. 8806402 10.1007/BF00019459

[B27] Müller, K.M., Sheath, R.G., Vis, M.L., Crease, T.J., and Cole, K.M. (1998) Biogeography and systematics of *Bangia* (Bangiales, Rhodophyta) based on the Rubisco spacer, *rbcL* gene and 18S rRNA gene sequences and morphometric ana­lyses. 1. North America. Phycologia 37: 195–207.

[B28] Nagano, Y., Kimura, K., Kobayashi, G., and Kawamura, Y. (2021) Genomic diversity of 39 samples of *Pyropia* species grown in Japan. PLoS One 16: e0252207. 34106965 10.1371/journal.pone.0252207 PMC8189503

[B29] Nguyen, L.T., Schmidt, H.A., von Haeseler, A., and Minh, B.Q. (2015) IQ-TREE: A fast and effective stochastic algorithm for estimating maximum-likelihood phylogenies. Mol Biol Evol 32: 268–274. 25371430 10.1093/molbev/msu300 PMC4271533

[B30] Nibert, M.L., Vong, M., Fugate, K.K., and Debat, H.J. (2018) Evidence for contemporary plant mitoviruses. Virology 518: 14–24. 29438872 10.1016/j.virol.2018.02.005 PMC6668999

[B31] Ninomiya, A., Urayama, S., Suo, R., Itoi, S., Fuji, S., Moriyama, H., and Hagiwara, D. (2020) Mycovirus-induced tenuazonic acid production in a rice blast fungus *Magnaporthe oryzae*. Front Microbiol 11: 1641. 32765467 10.3389/fmicb.2020.01641 PMC7379127

[B32] Ogata, E. (1970) On a new algal culture medium SWM-III. Bull Jpn Soc Phycol 18: 171–173 (in Japanese).

[B33] Polashock, J.J., and Hillman, B.I. (1994) A small mitochondrial double-stranded (ds) RNA element associated with a hypovirulent strain of the chestnut blight fungus and ancestrally related to yeast cytoplasmic T and W dsRNAs. Proc Natl Acad Sci U S A 91: 8680–8684. 7521532 10.1073/pnas.91.18.8680 PMC44670

[B34] Polashock, J.J., Bedker, P.J., and Hillman, B.I. (1997) Movement of a small mitochondrial double-stranded RNA element of *Cryphonectria parasitica*: ascospore inheritance and implications for mitochondrial recombination. Mol Gen Genet 256: 566–571. 9413441 10.1007/s004380050602

[B35] Powell, M.L. (2010) Translational termination–reinitiation in RNA viruses. Biochem Soc Trans 38: 1558–1564. 21118126 10.1042/BST0381558

[B36] Roossinck, M.J. (2010) Lifestyles of plant viruses. Philos Trans R Soc B 365: 1899–1905. 10.1098/rstb.2010.0057 PMC2880111 20478885

[B37] Roossinck, M.J. (2011) The good viruses: Viral mutualistic symbioses. Nat Rev Microbiol 9: 99–108. 21200397 10.1038/nrmicro2491

[B38] Roossinck, M.J. (2012) Persistent plant viruses: molecular hitchhikers or epigenetic elements? In *Viruses: Essential Agents of Life*. Witzany, G. (ed.) New York, NY: Springer, pp. 177–186.

[B39] Roossinck, M.J. (2015) Metagenomics of plant and fungal viruses reveals an abundance of persistent lifestyles. Front Microbiol 5: 767. 25628611 10.3389/fmicb.2014.00767 PMC4290624

[B40] Sadeghi, M., Tomaru, Y., and Ahola, T. (2021) RNA viruses in aquatic unicellular eukaryotes. Viruses 13: 362. 33668994 10.3390/v13030362 PMC7996518

[B41] Safari, M., Ferrari, M.J., and Roossinck, M.J. (2019) Manipulation of aphid behavior by a persistent plant virus. J Virol 93: e01781-18. 30760572 10.1128/JVI.01781-18 PMC6475794

[B42] Saunders, G.W., and Kraft, G.T. (1994) Small-subunit rRNA gene sequences from representatives of selected families of the Gigartinales and Rhodymeniales (Rhodophyta). 1. Evidence for the Plocamiales ord. nov. Can J Bot 72: 1250–1263.

[B43] Scholthof, K.B.G., Adkins, S., Czosnek, H., Palukaitis, P., Jacquot, E., Hohn, T., et al. (2011) Top 10 plant viruses in molecular plant pathology. Mol Plant Pathol 12: 938–954. 22017770 10.1111/j.1364-3703.2011.00752.x PMC6640423

[B44] Short, S.M., Staniewski, M.A., Chaban, Y.V., Long, A.M., and Wang, D. (2020) Diversity of viruses infecting eukaryotic algae. Curr Issues Mol Biol 39: 29–62. 32073403 10.21775/cimb.039.029

[B45] Urayama, S., Takaki, Y., and Nunoura, T. (2016) FLDS: A comprehensive dsRNA sequencing method for intracellular RNA virus surveillance. Microbes Environ 31: 33–40. 26877136 10.1264/jsme2.ME15171 PMC4791113

[B46] Urayama, S., Takaki, Y., Nishi, S., Yoshida-Takashima, Y., Deguchi, S., Takai, K., and Nunoura, T. (2018) Unveiling the RNA virosphere associated with marine microorganisms. Mol Ecol Resour 18: 1444–1455. 30256532 10.1111/1755-0998.12936

[B47] Urayama, S., Takaki, Y., Hagiwara, D., and Nunoura, T. (2020) dsRNA-seq reveals novel RNA virus and virus-like putative complete genome sequences from *Hymeniacidon* sp. sponge. Microbes Environ 35: ME19132. 32115438 10.1264/jsme2.ME19132 PMC7308569

[B48] Walker, P.J., Siddell, S.G., Lefkowitz, E.J., Mushegian, A.R., Adriaenssens, E.M., Dempsey, D.M., et al. (2020) Changes to virus taxonomy and the statutes ratified by the international committee on taxonomy of viruses (2020). Arch Virol 165: 2737–2748. 32816125 10.1007/s00705-020-04752-x

[B49] Xu, P., Chen, F., Mannas, J.P., Feldman, T., Sumner, L.W., and Roossinck, M.J. (2008) Virus infection improves drought tolerance. New Phytol 180: 911–921. 18823313 10.1111/j.1469-8137.2008.02627.x

[B50] Yang, L.E., Deng, Y.Y., Xu, G.P., Russell, S., Lu, Q.Q., and Brodie, J. (2020) Redefining *Pyropia* (Bangiales, Rhodophyta): four new genera, resurrection of *Porphyrella* and description of *Calidia pseudolobata* sp. nov. from China. J Phycol 56: 862–879. 32196675 10.1111/jpy.12992

