# Supplementary material for: Detection and Characterization of RNA Viruses in Red Macroalgae (Bangiaceae) and Their Food Product (Nori Sheets)
Source: Microbes Environ. 2022 Jun 10;37(5):ME21084. doi: 10.1264/jsme2.ME21084 (PMC9763034; doi:10.1264/jsme2.ME21084)
Supplement: Supplementary file 1 — Supplementary Material [file 37_21084_s1.pdf]

Supplementally Table 1. Similarities (%) in the partial RdRp gene of NMV1 (up-right) and NMV2 (down-left) among the conchocelis samples.

	C1	C2	C3	C4	C5	C6	C7	C8	C9	C10	C11	C12	C13	C14	C15	C16	C17	C18	C19	C20	C21	C22	C23	C24	C25	C26	C27	C28	C29	C30	C31	C32
**C 1**		-	92.3	-	-	-	99.8	-	99.9	89.7	99.5	99.5	90.6	99.5	99.4	99.4	99.7	99.8	99.8	99.4	99.9	99.8	92.2	99.9	99.8	99.8	99.8	99.9	99.9	99.9	99.8	99.8
**C2**	-		-	-	-	-	-	-	-	-	-	-	-	-	-	-	-	-	-	-	-	-	-	-	-	-	-	-	-	-	-	-
**C3**	-	-		-	-	-	92.3	-	92.25	91.00	92.4	92.4	91.1	92.4	92.5	92.7	92.1	92.5	92.5	92.7	92.4	92.4	99.7	92.4	92.3	92.4	92.4	92.4	92.4	92.4	92.5	92.3
**C4**	-	-	-		-	-	-	-	-	-	-	-	-	-	-	-	-	-	-	-	-	-	-	-	-	-	-	-	-	-	-	-
**C5**	-	-	-	-		-	-	-	-	-	-	-	-	-	-	-	-	-	-	-	-	-	-	-	-	-	-	-	-	-	-	-
**C6**	-	-	-	-	-		-	-	-	-	-	-	-	-	-	-	-	-	-	-	-	-	-	-	-	-	-	-	-	-	-	-
**C7**	99.3	-	-	-	-	-		-	99.9	90.2	99.6	99.6	91.1	99.6	99.5	99.5	99.7	99.8	99.8	99.5	99.9	100	92.2	99.9	99.7	99.8	99.8	99.7	99.8	99.9	99.8	99.8
**C8**	-	-	-	-	-	-	-		-	-	-	-	-	-	-	-	-	-	-	-	-	-	-	-	-	-	-	-	-	-	-	-
**C9**	99.7	-	-	-	-	-	99.1	-		90.2	99.6	99.6	91.1	99.6	99.6	99.6	99.8	99.9	99.9	99.6	100	99.9	92.2	100	99.8	99.9	99.9	99.8	99.9	100	99.9	99.9
**C10**	98.1	-	-	-	-	-	98.1	-	97.8		89.6	89.6	99.1	89.6	89.7	90	90	89.9	89.9	90	89.8	89.8	90.8	89.8	89.9	89.8	89.8	89.8	89.8	89.8	89.9	89.7
**C11**	99.4	-	-	-	-	-	99.9	-	99.1	98.0		99.8	90.5	99.8	99.7	99.3	99.5	99.5	99.5	99.3	99.6	99.5	92.5	99.6	99.5	99.5	99.5	99.4	99.4	99.6	99.5	99.5
**C12**	100	-	-	-	-	-	99.3	-	99.7	98.1	99.4		90.5	100	99.9	99.3	99.5	99.5	99.5	99.3	99.6	99.5	92.7	99.6	99.5	99.5	99.5	99.4	99.4	99.6	99.5	99.5
**C13**	99.0	-	-	-	-	-	98.5	-	98.7	97.1	98.5	99.0		90.5	90.6	90.9	90.9	90.8	90.8	90.9	90.7	90.7	90.9	90.7	90.8	90.7	90.7	90.7	90.7	90.7	90.8	90.6
**C14**	-	-	-	-	-	-	-	-	-	-	-	-	-		99.9	99.3	99.5	99.5	99.5	99.3	99.6	99.5	92.7	99.6	99.5	99.5	99.5	99.4	99.4	99.6	99.5	99.5
**C15**	99.4	-	-	-	-	-	99.9	-	99.1	98.2	99.8	99.4	98.5	-		99.2	99.4	99.4	99.4	99.2	99.5	99.4	92.8	99.5	99.4	99.4	99.4	99.3	99.3	99.5	99.6	99.4
**C16**	99.6	-	-	-	-	-	99.2	-	99.5	98.2	99.3	99.6	98.5	-	99.3		99.4	99.6	99.6	100	99.5	99.4	92.4	99.5	99.4	99.4	99.4	99.3	99.3	99.5	99.4	99.4
**C17**	-	-	-	-	-	-	-	-	-	-	-	-	-	-	-	-		99.7	99.7	99.4	99.8	99.7	92.0	99.8	99.6	99.7	99.7	99.6	99.7	99.8	99.7	99.7
**C18**	99.8	-	-	-	-	-	99.1	-	99.6	98.1	99.2	99.8	98.8	-	99.2	99.6	-		100	99.6	99.9	99.8	92.4	99.9	99.8	99.8	99.8	99.7	99.7	99.9	99.8	99.8
**C19**	99.7	-	-	-	-	-	99.1	-	99.5	98	99.1	99.7	98.7	-	99.1	99.5	-	99.9		99.6	99.9	99.8	92.4	99.9	99.8	99.8	99.8	99.7	99.7	99.9	99.8	99.8
**C20**	99.6	-	-	-	-	-	99.2	-	99.5	98.2	99.3	99.6	98.5	-	99.3	100	-	99.6	99.5		99.5	99.4	92.4	99.5	99.4	99.4	99.4	99.3	99.3	99.5	99.4	99.4
**C21**	99.5	-	-	-	-	-	99.1	-	99.4	98.1	99.2	99.5	98.5	-	99.2	99.9	-	99.5	99.6	99.9		99.9	92.3	100	99.9	99.9	99.9	99.8	99.8	100	99.9	99.9
**C22**	99.8	-	-	-	-	-	99.3	-	99.6	97.9	99.4	99.8	99.1	-	99.4	99.4	-	99.7	99.6	99.4	99.3		92.3	99.9	99.8	99.8	99.8	99.7	99.7	99.9	99.8	99.8
**C23**	99.8	-	-	-	-	-	99.3	-	99.6	97.9	99.4	99.8	99.1	-	99.4	99.4	-	99.7	99.6	99.4	99.3	100		92.3	92.2	92.3	92.3	92.3	92.3	92.3	92.4	92.2
**C24**	99.9	-	-	-	-	-	99.2	-	99.7	98.00	99.3	99.9	99.1	-	99.3	99.5	-	99.7	99.7	99.5	99.4	99.9	99.9		99.9	99.9	99.9	99.8	99.8	100	99.9	99.9
**C25**	99.9	-	-	-	-	-	99.2	-	99.7	98.00	99.3	99.9	98.9	-	99.3	99.5	-	99.7	99.7	99.5	99.4	99.7	99.7	99.8		99.8	99.8	99.7	99.7	99.9	99.8	99.8
**C26**	100	-	-	-	-	-	99.3	-	99.7	98.1	99.4	100	99.0	-	99.4	99.6	-	99.8	99.7	99.6	99.5	99.8	99.8	99.9	99.9		99.8	99.7	99.7	99.9	99.8	99.8
**C27**	99.8	-	-	-	-	-	99.1	-	99.6	97.9	99.2	99.8	98.8	-	99.2	99.4	-	99.7	99.7	99.4	99.5	99.7	99.7	99.7	99.7	99.8		99.7	99.7	99.9	99.8	99.8
**C28**	99.7	-	-	-	-	-	99.4	-	99.5	98.2	99.5	99.7	98.7	-	99.5	99.5	-	99.6	99.5	99.5	99.4	99.6	99.6	99.7	99.7	99.7	99.6		100	99.8	99.7	99.7
**C29**	99.9	-	-	-	-	-	99.2	-	99.7	98.00	99.3	99.9	98.9	-	99.3	99.5	-	99.7	99.8	99.5	99.6	99.7	99.7	99.8	99.8	99.9	99.9	99.7		99.8	99.7	99.7
**C30**	100	-	-	-	-	-	99.3	-	99.7	98.1	99.4	100	99.0	-	99.4	99.6	-	99.8	99.7	99.6	99.5	99.8	99.8	99.9	99.9	100	99.8	99.7	99.9		99.9	99.9
**C31**	99.9	-	-	-	-	-	99.2	-	99.7	98.00	99.3	99.9	98.9	-	99.3	99.5	-	99.7	99.8	99.5	99.6	99.7	99.7	99.8	99.8	99.9	99.9	99.7	100	99.9		99.8
**C32**	100	-	-	-	-	-	99.3	-	99.7	98.1	99.4	100	99.0	-	99.4	99.6	-	99.8	99.7	99.6	99.5	99.8	99.8	99.9	99.9	100	99.8	99.7	99.9	100	99.9	

Sequence similarity of NMV2

Sequence similarity of NMV1
